# Effects of “Reduced” and “Business-As-Usual” CO_2_ Emission Scenarios on the Algal Territories of the Damselfish *Pomacentrus wardi* (Pomacentridae)

**DOI:** 10.1371/journal.pone.0131442

**Published:** 2015-06-29

**Authors:** Dorothea Bender, Connor Michael Champ, David Kline, Guillermo Diaz-Pulido, Sophie Dove

**Affiliations:** 1 School of Biological Sciences & Global Change Institute, University of Queensland, Queensland, Australia; 2 ARC Centre for Excellence for Coral Reef Studies, University of Queensland, Queensland, Australia; 3 Queensland Brain Institute, University of Queensland, Queensland, Australia; 4 Griffith School of Environment and Australian Rivers Institute, Griffith University, Queensland, Australia; College of Charleston, UNITED STATES

## Abstract

Turf algae are a very important component of coral reefs, featuring high growth and turnover rates, whilst covering large areas of substrate. As food for many organisms, turf algae have an important role in the ecosystem. Farming damselfish can modify the species composition and productivity of such algal assemblages, while defending them against intruders. Like all organisms however, turf algae and damselfishes have the potential to be affected by future changes in seawater (SW) temperature and *p*CO_2_. In this study, algal assemblages, in the presence and absence of farming *Pomacentrus wardi* were exposed to two combinations of SW temperature and *p*CO_2_ levels projected for the austral spring of 2100 (the B1 “reduced” and the A1FI “business-as-usual” CO_2_ emission scenarios) at Heron Island (GBR, Australia). These assemblages were dominated by the presence of red algae and non-epiphytic cyanobacteria, i.e. cyanobacteria that grow attached to the substrate rather than on filamentous algae. The endpoint algal composition was mostly controlled by the presence/absence of farming damselfish, despite a large variability found between the algal assemblages of individual fish. Different scenarios appeared to be responsible for a mild, species specific change in community composition, observable in some brown and green algae, but only in the absence of farming fish. Farming fish appeared unaffected by the conditions to which they were exposed. Algal biomass reductions were found under “reduced” CO_2_ emission, but not “business-as-usual” scenarios. This suggests that action taken to limit CO_2_ emissions may, if the majority of algae behave similarly across all seasons, reduce the potential for phase shifts that lead to algal dominated communities. At the same time the availability of food resources to damselfish and other herbivores would be smaller under “reduced” emission scenarios.

## Introduction

Epilithic turf algal communities are composed of filamentous eukaryotic algae, diatoms, cyanobacteria, as well as juvenile macroalgae [1]. They are ubiquitous on coral reefs and provide food for fish, e.g. damselfish, and invertebrates such as gastropods, sea urchins and crustaceans [1,2]. The continuous cropping by such herbivores is the reason for their low standing stock [3] that can only be sustained by their high net productivity [4–10]. Consequently, the turnover rate of these communities is very high (4–12 days, [11]). The importance of such communities is highlighted by the fact that turf-algae-dominated damselfish territories can occupy up to 90% of the reef substratum [12,13], suggesting that the fate of turf algae within such algal assemblages will at least in part determine the impact of future conditions on coral reefs.

Generally speaking, the productivity of turf algae within the territories of damselfishes has been found to be higher than outside damselfish territories [11,14,15]. Several reasons for increased productivity within damselfish territories have been offered that include: cropping, algae to sizes that favor exponential growth [11,14], nutrient provisioning [11,12,16,17] or selective removal of less productive algae [11,14]. The contribution each of these factors makes to the increased productivity inside damselfish territories will most likely vary between fish species.

The test species of this study, *Pomacentrus wardi* Whitley (Ward’s damsel), is common to Heron Island Reef and other parts of the Great Barrier Reef (GBR) [13,18,19] and its distribution seems to be limited to inshore and mid-shelf reefs on the central GBR [20]. *P*. *wardi* has been described to be omnivorous to some extent, yet mostly herbivorous [13]. Despite a high variability between areas and between territories within coral reefs, generally the cover of red algae (*Polysiphonia*, *Gelidiopsis*, *Jania*, *Laurencia* and the group of corticated red algae) and detritus is elevated inside territories compared to areas that are not farmed [13,19]. The variability in turf species composition observed between areas and within distinct reefs has been attributed to the farming activity of the fish, the benthic assemblages in the different reef areas and the site selection of the fish [13,19,21–23]. In general, the differences in species composition between areas outside the territory can be maintained as damselfishes defend their territories against other fishes and herbivorous invertebrates [21,24–26].

The habitat of damselfish and their algal territories, coral reefs, are threatened by elevated temperatures and ocean acidification [27]. Past studies had the tendency to focus on the effects future seawater conditions may have on phase shifts in these ecosystems, specifically in accelerating potential coral-algal phase shifts [28]. In these studies, algae are usually viewed as detrimental to reefs, because of their ability to outcompete corals due to a variety of competitive mechanisms, which vary in effectiveness [29]. However, turf algae are also an essential component to reef ecology due to their high productivity [30]. Like most other organisms, turf algae are likely to be affected by changing conditions. Two previous studies have focused on the settlement and subsequent cover of temperate turf algae dominated by one brown algal genus (*Feldmannia* sp.), which responded with increased biomass and cover to decreases in pH and elevations in temperatures. However, the two factors synergistically increased cover but not biomass [31,32].

The relative abundance of species in future algal assemblages will be determined, at least in part, by their performance under the changed conditions. Algae differ in mechanisms used to concentrate CO_2_ at the site of fixation. The red algal group is often the most dominant in damselfish territories and its form of the CO_2_ fixing enzyme RubisCO is generally more efficient in discriminating between CO_2_ and O_2_ than that of green or brown algae [33,34]. This ability enhances energy conservation, making carbon-concentrating mechanisms (CCMs) less important and, depending on the niche inhabited by the algae, even unnecessary for some red algae [35,36]. Cyanobacteria, most green and brown algae and the red algae that possess a CCM, may potentially be able to down-regulate CCM activity as the CO_2_ concentration in their medium is elevated (e.g. [37] [38–40]). This down regulation could lead to energy conservation in the respective species and render them more competitive against some of the red algal species that do not posses a CCM and therefore benefit in a more direct way from increased CO_2_ availability [38–40].

The differential responses of algal communities to changes in CO_2_ concentration and SW temperature are currently understudied [38]. The combined effects of ocean acidification and increasing temperature have in particular received little attention, despite some observations reporting antagonistic effects: Increasing temperature causes RubisCO to be less efficient at fixing carbon as its ability to discriminate CO_2_ from O_2_ decreases and photorespiration is favored e.g. [41,42]. However, an increase in CO_2_ concentration could increase carbon fixation and therefore productivity [31,32,43–46]. From these observations, predictions can be made, but these remain to be tested. The present study is an attempt to assess possible effects of future conditions on the composition, productivity and growth of algal communities within damselfish territories.

In addition to a possible increase in the algal production of damselfish farms, the fish themselves are also likely to be affected by ocean acidification and elevated temperature. Ocean acidification leads to potential changes in behavior in damselfish likely caused by alterations in (1) the olfactory system through interference with neurotransmitter function and in (2) the visual or auditory system, leading to boldness and failure to recognize predators [47–49]. These effects on damselfish can influence prey selectivity [50] and therefore potentially the composition of algal turfs.

This present study aimed to investigate the potential impact of business-as-usual (A1FI) and “reduced” (B1) CO_2_ emission scenarios on turf algal assemblages within territories of the damselfish *P*. *wardi*. All future predictions regarding the effects of increased atmospheric CO_2_ on the oceans involve simultaneous increases in ocean temperature and acidification [51]. In the present study, it was hypothesized that algal territories would be affected differently at the end-of-the-century by “reduced” (B1) versus “business-as-usual” (A1FI) ocean conditions associated with differential rates of CO_2_ emission through to 2050 [50]. It was further hypothesized that the response would be modified by the presence or absence of farming *P*. *wardi*. As the experiment aimed to determine the response of these organisms to future conditions, appropriate anomalies assigned to B1 (“reduced” CO_2_ emission scenarios) and A1FI (“business-as usual” CO_2_ emission scenarios) SW temperature and CO_2_ concentrations were applied as offsets to a present-day austral spring baseline to generate contrasting conditions that are representative of effective and ineffective actions to stem the build-up of CO_2_ in the atmosphere.

## Materials and Methods

Experiments were carried out with permission from the University of Queensland Ethics Committee (AEC approval number GCI/184/10), the Great Barrier Reef Marine Park Authority (permit number UQ002/2010) and the Queensland Fisheries Service (permit number 140650).

To assess the effect of elevated temperature and ocean acidification on damselfish turf algal communities, the fish and their algal assemblages were subjected to two different scenario treatments [51], and a control or present-day (PD) scenario. The scenarios were applied as offset for both temperature and *p*CO_2_ to the PD. The treatment conditions were as follows: PD treatment (*p*CO_2_ of 387 μatm and reef-flat temperature of 22.4°C), B1 or “reduced” CO_2_ emission scenario (+220 μatm, +2.3°C), and the A1FI or “business-as-usual” CO_2_ scenario (+583 μatm, +4.1°C). CO_2_ was injected on demand using solenoid valves into 8,000 L sumps where the water was also heated. The incoming water at Heron Island Research Station (HIRS) is stored in a large reservoir, therefore the *p*CO_2_ and temperature have a potential to be modified. To counter these effects, PD set-points were modified to mirror seasonally appropriate weekly *p*CO_2_ and temperature averages measured on the reef flat. For the B1, A1FI and PD treatment, the *p*CO_2_ was adjusted by adding or removing CO_2_ from the water. The latter was achieved by passing air through soda lime and then into the respective sump. For the actual *p*CO_2_ values obtained in the respective treatments see Table 1. Temperature was adjusted to set-points with the use of industrial heater-chillers (HWPO17-1BB; Rheem). Notably average temperatures achieved under A1FI (26.6 ± 1.33°C, mean ± SD) was less than the maximum monthly mean (MMM) of 27.3°C established for Heron Island [52]. A maximum of 4°C above present-day conditions applied on a high latitude reef such as Heron Island where the annual temperature variation is ~7°C will not necessarily lead to anomalous temperatures for specific times of year or the life time of the organisms involved. For this specific study, we decided to monitor the response to future spring conditions acknowledging that differential responses may be observed in other seasons. Whilst the *p*CO_2_ in the PD treatment was slightly elevated from the monitored reef site, it was very different from the A1FI and B1 treatments. Differences observed between the reef flat site and PD treatment were roughly 30 μatm, a magnitude of difference that could easily be observed over a reef flat and the larger area the samples were collected from.

**Table 1:** Water chemistry data.

	scenario			
	PD	B1	A1FI	HIRS reef flat
temperature (°C)	22.48 ± 0.72	24.76 ± 0.94	26.61 ± 1.33	22.70 ± 1.20
pH_Total_	8.17 ± 0.005	8.01 ± 0.006	7.85 ± 0.007	8.11 ± 0.11
*p*CO_2_ (μatm)	386.96 ± 35.04	607.52 ± 48.49	970.03 ± 65.53	355.38 ± 12.06
total alkalinity day (μmol Eq L^-1^)	2235 ± 39	2226 ± 45	2223 ± 51	2246.6 ± 35.4 (mean of day and night)
total alkalinity night (μmol Eq L^-1^)	2226 ± 20	2225 ± 21	2225 ± 19	
ammonium (mg L^-1^)	0.0036 ± 0.0009	0.0044 ± 0.0021	0.0048 ± 0.002	0.0085 ± 0.001

Mean temperature (recorded in 10 min intervals), pH_Total_ (recorded 5 times daily), total alkalinity (measured once a week at midday and midnight), and ammonium concentration measured in aquaria (15^th^ October 2010) and on the reef flat about 100 m from the seawater intake (daily). *p*CO_2_ was measured in the sumps (logged continuously every 3 min) and calculated in CO2SYS (developed by E. Lewis and W.R. Wallace) for the reef flat based on twice daily alkalinity and salinity sampling, and continuous temperature and pH monitoring at 10 minute intervals (see [53] for details). PD = present-day scenario, B1 = “reduced” emission scenario, A1FI = “business-as-usual” scenario. Mean ± SD for all values.

Damselfish, *Pomacentrus wardi*, along with pieces of their algal territory were collected from Shark Bay, HIRS, Australia (23°26’S, 151°52’W). Fifteen fish and six pieces of their algal territory (90 pieces in total) were collected and separated into three scenario treatments of five fish each. The damselfish were randomly selected, the only constraints being a distance of 1.5 m between fish territories and a body length of ~6 cm. Before collection, the fish were observed whilst they fed on their algal turfs. The feeding areas were marked and the fish caught using hand nets and a diluted clove oil solution (< 5%, [54]). Subsequently, the algal assemblages attached to their calcium carbonate substrate were collected using hammer and chisel. Each was cut into a circle 2.5 cm in diameter and cleaned of animals (mostly polychaetes and crustaceans). The protocol for catching and holding the fish was approved by the University Animal Ethics Committee of the University of Queensland (Permit Number: GCI/184/10) and the Queensland Government Department of Primary Industries and Fisheries (Permit Number: 140650). The extraction of *P*. *wardi* as well as its algal assemblages was covered under the Limited Impact Accreditation No. UQ002/2010.

**Fig 1:**
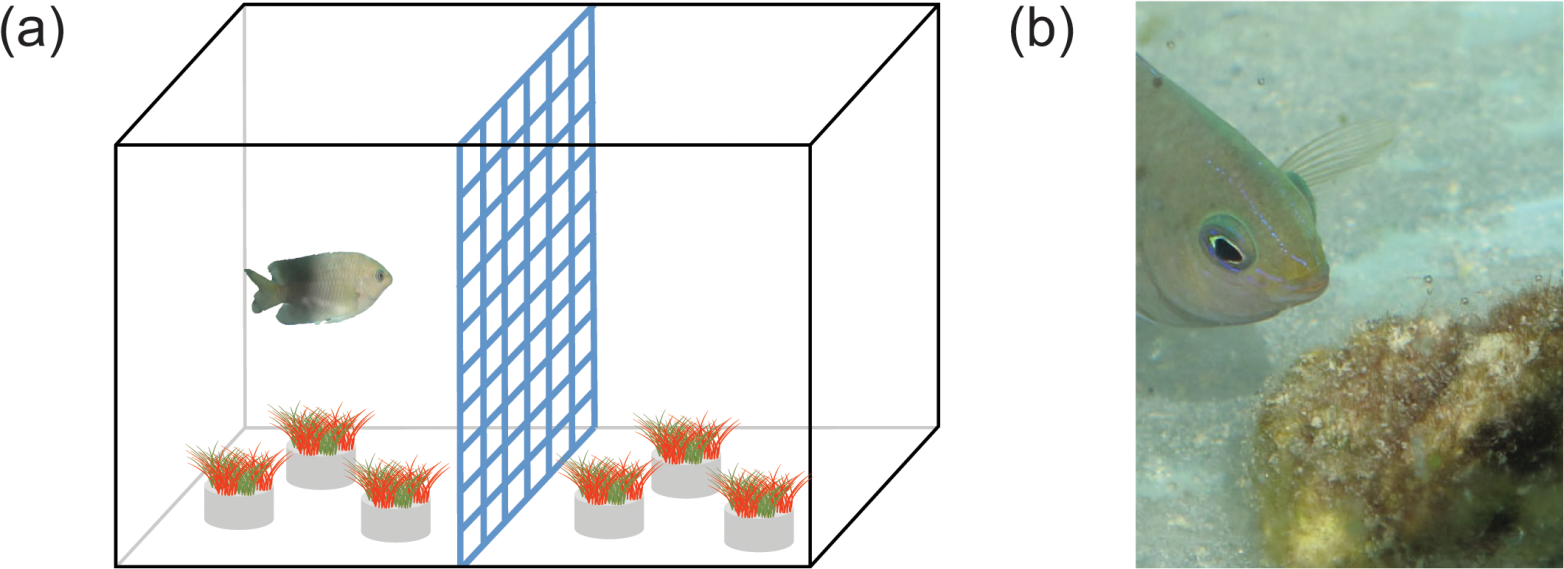
The experimental set-up. (a) Compartmentalization of aquaria (n = 15 aquaria with n = 5 per scenario) and (b) *P*. *wardi* with an algal assemblage. Fish were placed into one half of the aquarium with 3 of their algal assemblages. The other half of the aquarium also contained 3 algal assemblages but was fenced off by plastic grating (grate size 1 cm^2^). The grating was cleaned frequently to allow for efficient exchange of water and nutrients between tank halves which was also aided by one powerhead per aquarium. Photo: D. Bender

The experimental aquaria were divided in half by a plastic grating, grid size 1 cm^2^, that allowed for water, nutrient and particle movement between the two sides of the tank, but restricted the fish to one side for the duration of the experiment (Fig 1). By separating the tanks into halves in this manner, the effects of the distinct scenario treatments on algae turfs could be investigated in the presence and absence of grazing/farming while potential nutrient enrichment from fish excretion was applied across all treatments. To ensure nutrient redistribution throughout the tank, water circulation and distribution of particles and excreted compounds was aided by pumps (Clearpond Infiniti 800). The glass aquaria and their lids were covered with 0.3 neutral density filters (LEE International) in order to simulate light intensities at 2–0.5 m depth on the reef flat. The filters also minimized potential light stress on algae and fish due to the outdoors location of the set-up. The average daily maximum photon flux inside the aquaria was 624 μmol m^-2^ s^-1^.

Three turf algal samples were put in each half of the 60 L glass aquaria (n = 15 glass aquaria, 5 per treatment) and one fish was placed into one half. Fish were placed with their own specific algal samples taken from their individual territories. Additionally, the fish were provided with tubes as shelter. The fish started feeding and defending their territories within the first 24 h in their aquaria, and maintained this behavior throughout the experiment; showing that they got used to their new environment.

The fish and their algal assemblages were acclimated to the treatment conditions for 3 days, during which the treatments were mixed with incoming reef-flat SW so that they only received 50% of the respective *p*CO_2_ and temperature treatment. This relatively short acclimation period seemed appropriate given that organisms living on Heron Island reef flat experience relatively steep diurnal temperature and pH changes [e.g. 52,55] and that they would therefore experience even faster rates of change. After the acclimation period, the fish and algal communities were subjected to full treatment conditions for the experimental period of four weeks (austral spring: 15 September 2010 to 15 October 2010) in order to allow for a minimum of 2 turnover cycles of the turf algal assemblages [11]. The fish were closely monitored during the acclimation period and throughout the entire experiment for signs of stress or illness, i.e. changes in color or behavior. However, no such changes were observed.

The total alkalinity of the water inside each of the tanks was sampled weekly at midday and midnight (Table 1) and was measured using a Mettler Toledo titrating system (T50) by Gran titration after [56] using the method described in [52]. Each water sample was analysed in triplicate. Temperature loggers (HOBO Data Loggers, Onset) were placed in the aquaria to monitor temperature (Table 1). The in and out flow of the water was kept constant at 0.75 L min^-1^, leading to the turnover of water within aquaria every 75 min. The mean salinity on the reef flat during the experimental period was 35.2 ± 0.28 (mean ± SE).

The turf algal species composition expressed as percent cover was assessed using a 1 cm^2^ quadrat divided into 25 squares before and after the experimental period of four weeks. The most dominant genus or species in each of the 25 squares was scored under a dissection microscope to estimate the relative abundance of algal taxa. If necessary, algal genera were confirmed using a compound microscope. Fish grazing effects and absence of reproductive structures of some algal species hindered complete identification to species level.

In order to investigate treatment effects on turf algal metabolism, the rates of net oxygen production and consumption (respirometry) were determined. During the O_2_ flux measurements the maximum net productivity (P_nmax_) and dark respiration were measured [57]. Light curves provided a saturation irradiance of 700 ± 55 μmol m^-2^ s^-1^. The dark period (photon flux 0 μmol m^-2^ s^-1^) was followed by 15 min of maximum photon flux at 900 μmol m^-2^ s^-1^ (Ocean Light T5 MH combo 150 W, with 2x24 Ocean Blue Actinic, Aqua-Medic of North America, LLC), equivalent to an average mid-day summer photon flux of 1000 μmol m^-2^ s^-1^ on Heron Island reef-flat [58]. A 5 min light and photosynthesis induction occurred between dark respiration and P_nmax_ measurement. The respirometry data was normalized to biomass of the whole sample (mg ash free dry weight, mg_AFDW_, the difference between dry weight after 48 h of drying and the weight after incineration). All measurements were conducted in the respective experimental treatment water.

The algal biomass was established separately for the endolithic and epilithic (turf) communities at the end of the experiment. In order to do this, the turf community was scraped off the calcium carbonate substrate using a scalpel. Adhering or incorporated calcium carbonate was subsequently dissolved in 30 ml of 1 M HCl solution for 24 h. The left-over calcium carbonate block containing the endolithic community was then dissolved in 160 ml of 1M HCl for up to 5 days until all calcium carbonate was dissolved. The calcium carbonate free turf (epilithic) and endolithic algae samples were then filtered onto pre-weighed glass filter paper (Whatman, GF/F, 0.7 μm), which had been combusted at 550°C for 5 h. During the filtrations, 30 and 260 ml of distilled water were used for the turf and endolithic community samples respectively. The samples on the filters were then dried to stable weight in an oven for 48 h at 60°C. Finally, the samples were combusted in a muffle furnace at 550°C for 5 h. The ash free dry weight was calculated as the difference between dry weight after 48 h of drying and the weight after incineration. The biomass was then normalized to the area of the sample (~ 4.91 cm^2^).

Seawater samples were taken from every tank (n = 15) on the final treatment day (15^th^ October 2010) to ascertain the concentration of ammonia in the treatment tanks. The samples were filtered (0.45 μm), immediately frozen and then analyzed to a precision of 0.001 mg L^-1^ (LACHAT 8500QC flow injection system, Queensland Health, Forensic and Scientific Services). Ammonium levels were 0.0036 ± 0.0009 mg L^-1^ (mean ± SD) in PD, 0.0044 ± 0.0021 mg L^-1^ in B1 and 0.0048 ± 0.002 mg L^-1^ in A1FI treatments, while the reef flat water contained 0.0085 ± 0.001 mg L^-1^ ammonium (Table 1).

The statistical analysis for the relative abundance of species data was conducted using PRIMER v6 software and PERMANOVA + add on (PRIMER-E Ltd). A two factorial (factors: treatment and herbivory, both fixed, and tanks nested within the interaction between treatment and herbivory) permutational analysis of variance (PERMANOVA) was run for all data based on a Bray Curtis Similarity with a dummy variable added [59]. The PERMANOVA analysis was run as a type III analysis (partial sum of squares type) using the method of permutation of residuals under a reduced model. Pair-wise tests were run after significant differences were found. For a 2D MDS plot on the basis of the Bray Curtis similarity with vector overlay based on a 0.3 Spearman correlation, only species occurring more than once were used (untransformed, plus dummy variable). It was noted that algal assemblages of different fish were highly variable at the beginning of the experiment. For this reason, the tanks were made the subject for a repeated measure ANOVA (analysis of variance). Fish (presence/absence) was then considered a within-subject factor, as was algal genus abundance (19 levels). To simplify the analysis, time was removed as a factor by selecting endpoint minus beginning (Δ%cover) as the main variable. This was achieved by determining the average cover of the 3 chips at the endpoint of the experiment and subtracting this from the initial cover averaged over 3 chips. Factorial ANOVAs were used on endpoint biomass and respirometry data, fixed factors being Scenario treatment and Herbivory. Assumption of homogeneity of data was tested using Levene’s test. If assumptions following transformation were not met, the significance level was restricted to P < 0.01 [60]. Post hoc analyses were performed using Newman-Keuls test. All ANOVAs were conducted using Statistica v9 (StatSoft). Highly correlated biomass (turf biomass, endolithic biomass, whole sample biomass) and respirometry (P_nmax_ and dark respiration) data were also analysed using PERMANOVA each, the fixed factors being Scenario and Herbivory.

## Results

No changes in species composition for turf algae exposed to water temperatures and ocean CO_2_ levels associated with present-day (PD), or end of century B1 (reduced CO_2_ emissions by 2050) or A1FI (business-as-usual CO_2_ emission) scenarios were observed when farming fish were present. However, significant differences in algal species composition among climate change scenarios were observed when fish were absent (Table 2, P(perm) = 0.002). Furthermore, irrespective of the presence of farming fish, algal turf biomass was smallest, but net and gross productivity per unit biomass were greatest under the B1 scenario.

**Table 2:** Statistical analysis of the species composition.

Source of variation	Sum of squares	df	Mean square	Pseudo-F	P(perm)	Conclusions/*Post hoc* test
**beginning**						
Sc	6692	2	3346	1.0	0.39	ns
H	1948	1	1948	0.6	0.66	ns
H x Sc	2770	2	1385	0.4	0.92	ns
TA(HxSc)	77488	24	3229	1.6	0.001*	species vary between tanks within scenarios
**end**						
Sc	10326	2	5163	1.3	0.18	ns
H	32872	1	32872	8.4	0.0001*	species composition with H ≠ without H
H x Sc	4174	2	2087	0.5	0.93	ns
TA(HxSc)	94232	24	3926	2.2	0.0001*	species vary between tanks within scenarios
**fish absent**						
Sc	11854	2	5927	2.2	0.002*	PD ≠ B1 = A1FI
**fish present**						
Sc	2938	2	1469	0.66	0.81	ns

Results of the statistical analysis using PERMANOVA for the species composition (relative abundance) of the turf algal assemblages before and after the experimental period (beginning/end) and separately for the samples kept in the presence and absence of the fish. ns = not significant,

* = P ≥ 0.05; H = herbivory; Sc = CO_2_ emission scenario; TA = tank; PD = present-day scenario.

### Herbivory effect on algal assemblage composition

**Fig 2:**
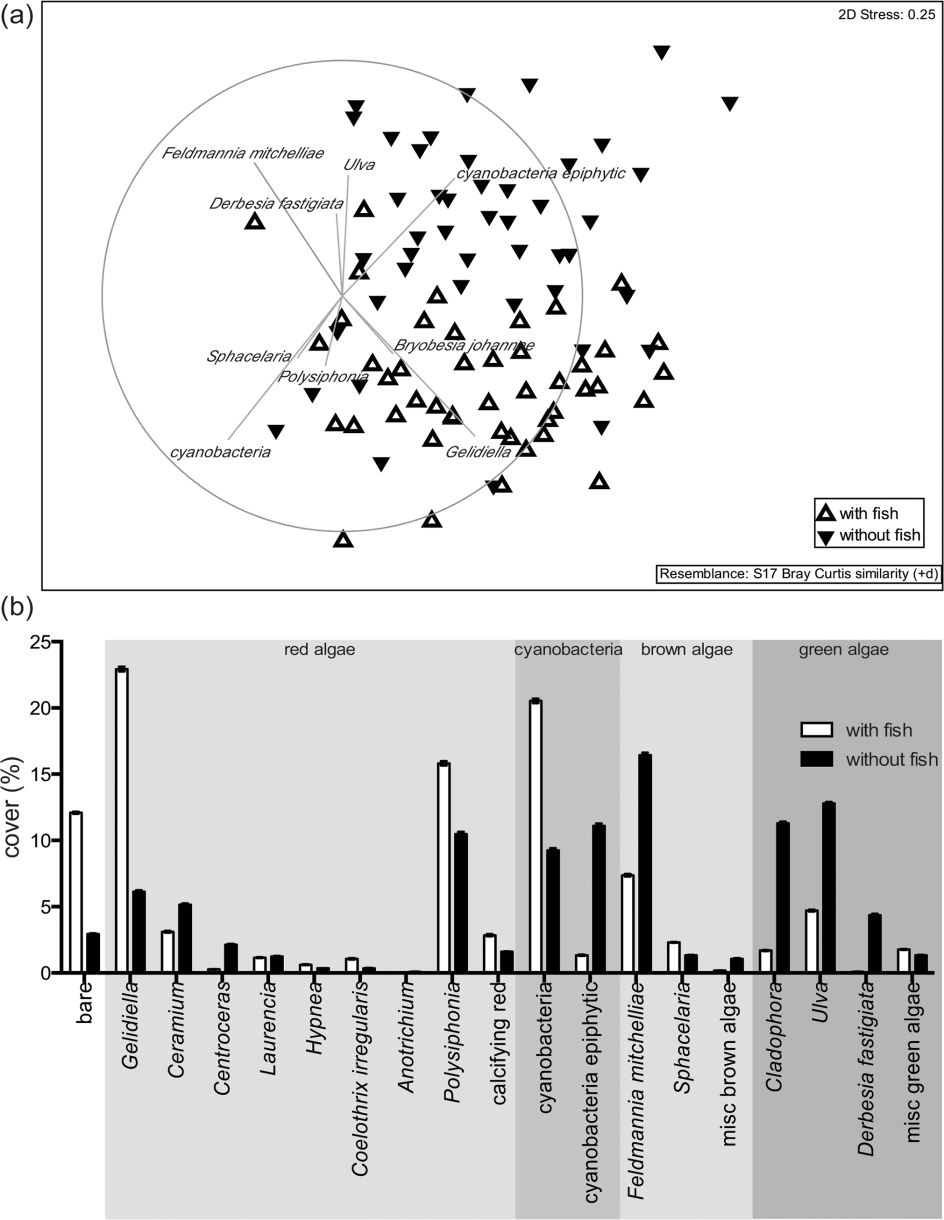
Turf algal species composition. The turf algal species composition in the presence and absence of farming damselfish at the end of the experimental period. (a) Two dimensional MDS plot on the basis of the Bray Curtis similarity with a vector overlay based on a 0.3 Spearman correlation. Only species occurring more than once were included (data were not transformed, plus dummy variable). (b) Percent cover of turf algal genera and groups (misc = miscellaneous). White triangles or bars = presence of damselfish farming, black triangles or bars = absence of damselfish farming for the duration of the experiment, n = 45 for each of the 2 herbivory treatments as data was pooled across CO_2_ emission scenarios.

**Fig 3:**
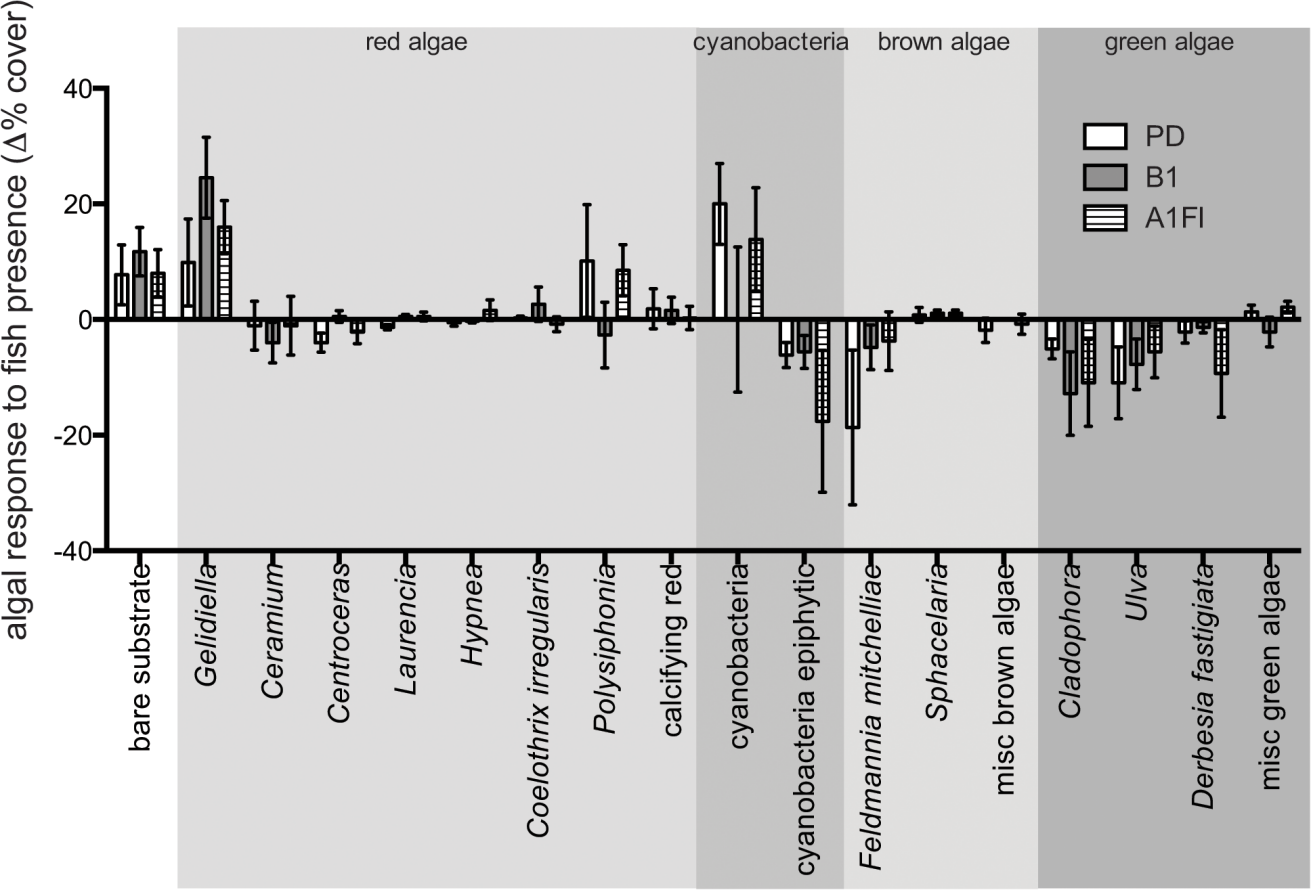
Percent cover of algae. The percent cover of algae in the presence minus the percent cover of algae in the absence of fish (mean ± SE), highlighting the impact of fish on algal turfs under present-day conditions (PD or present-day = open bars) and two CO_2_ emissions scenarios (B1 or “reduced” CO_2_ emission scenario = grey and A1FI or “business-as-usual” CO_2_ emission scenario = stripes). Only algae with a cover > 1% were included.

When collected from the field, the species composition of algal assemblages associated with distinct fish differed significantly from one another (Table 2, tank effect nested in the interaction of Herbivory and Scenario is significant, P(perm) = 0.001). Fish however, had a significant impact on community composition for either an analysis of end point only community structure or an analysis of percent change relative to initial cover (Δ%cover) (Figs 2 and 3, Table 2, P(perm) = 0.0001 and repeated measures ANOVA, F_(11, 132)_, P <0.00001). The presence of fish significantly promoted areas of bare substrate, certain red algae (e.g. *Gelidiella* and *Polysiphonia*) and cyanobacteria.

The MDS plot shows the effect the farming of *P*. *wardi* had on species composition with each symbol representing one sample (Fig 2A). The samples left without fish grazing for four weeks were dominated by cyanobacteria (Nostocales) that were epiphytic on eukaryotic turf algae (epiphytic cyanobacteria), and by filamentous brown (e.g. *Feldmannia mitchelliae)*, and green algae (e.g. *Cladophora*, *Ulva* and *Derbesia fastigiata)*. The samples subjected to farming, however, were dominated by corticated (*Gelidiella)* and filamentous red algae (*Polysiphonia)* and cyanobacteria (mainly Nostocales and Oscillatoriales). *Gelidiella*, *Polysiphonia*, cyanobacteria and epiphytic cyanobacteria were confirmed as the drivers of the difference between farmed and non-farmed algal assemblages by the repeated measures analysis (repeated measures ANOVA, Algal genus abundance x Herbivory, F_(11, 132)_, P <0.00001).

### Scenario effects on algal assemblage composition

In the endpoint data, turf algal species composition was not significantly affected by the interaction of factors (Herbivory x Scenario) or Scenario (Fig 2B, P(perm) = 0.93 and P(perm) = 0.18 respectively). Due to the variability amongst turf communities, there was the potential for subtle scenario treatment effects to be overlooked. The repeated measures analysis run with territories as subject and taking the starting point community composition into account, however, did not reveal any significant treatment effects (repeated measures ANOVA, F_(2, 12)_, P = 0.95). Despite this, a scenario effect was detected for endpoint data when turf assemblages kept in the presence and absence of fish were analyzed separately with permutational ANOVAs. In this case, a significant treatment effect was observed in the absence of fish farming. In the absence of fish, algal assemblages were significantly different under PD compared to B1 and A1FI conditions (Scenario: P(perm) = 0.002, Table 2, Fig 3), where *Feldmannia mitchelliae* and *Ulva* sp. responded negatively and *Cladophora* sp. and reacted positively to B1 and A1FI conditions. However, in the repeated measures analysis (using the change in % cover from endpoint minus beginning) based on samples kept in the absence of farming only, scenario treatment was not significant. This suggests that the distinction between PD and B1 or A1FI scenario may be due to the initial differences in the territories. Repeating the analysis, but limiting the data to *F*. *mitchelliae*, *Ulva* sp. and *Cladophora* sp. (as identified in the PERMANOVA analysis), does give a significant scenario effect for *F*. *mitchelliae*, where cover is greater in PD than in B1 or A1FI treatments (ANOVA, F_(2,12)_ = 7, P < 0.01).

### Effects on algal biomass

**Fig 4:**
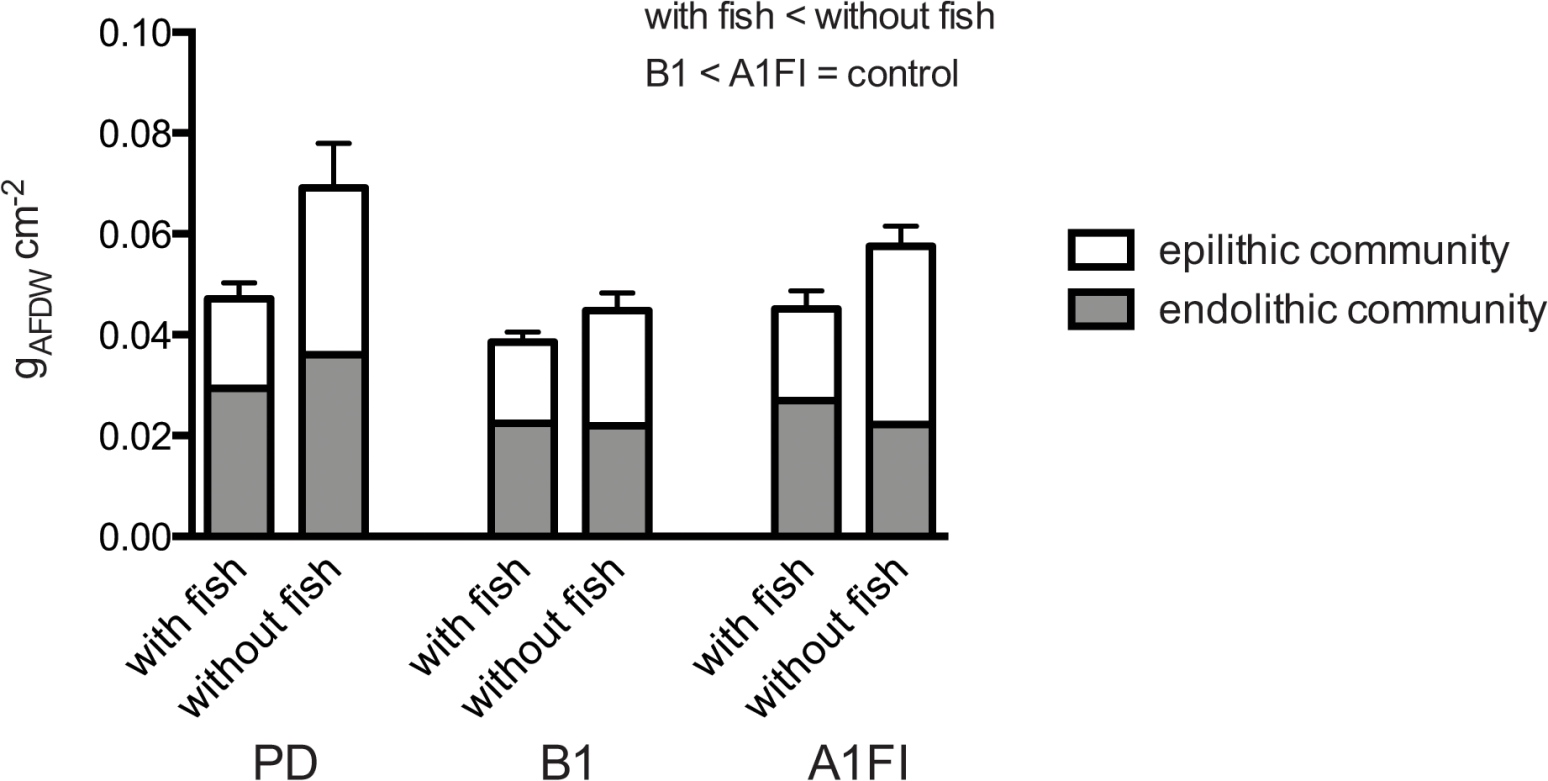
Algal biomass. The biomass of the turf algal communities under the different CO_**2**_ emission scenarios in the presence and absence of farming fish. Biomass in g_**AFDW**_ cm^-2^ of the epilithic turf community (white bars), the endolithic (grey bars) are shown (n = 15, mean ± SE of the whole sample). The results of the *Post-hoc* analysis highlight significant differences in whole sample biomass which was decreased in the presence of farming/grazing and under B1 scenarios compared to PD and A1FI scenarios. PD = present-day scenario, B1 = “reduced” CO_**2**_ emission scenario, A1FI = “business-as-usual” CO_**2**_ emission scenario.

The endolithic algal biomass at the end of the experiment was governed by the scenario treatments (Table 3, ANOVA, Scenario: F_(2,84)_, P = 0.02, Table 3) while fish farming and the interaction of herbivory and scenario had no significant effect (Table 3, ANOVA, Herbivory: F_(1, 84)_, P = 0.5; Herbivory x Scenario: F_(2, 84)_, P = 0.6). The PD samples had a significantly higher endolithic biomass than the B1 and A1FI turf communities (B1 biomass was reduced by 31%, A1FI biomass by 24%). The biomass of the epilithic turf community was in a similar range as the endolithic biomass (between 0.02 and 0.45 g_AFDW_ cm^-2^). In contrast, the epilithic algal biomass was significantly affected by both, scenario treatment and herbivory, but again, there was no interactive effect. While the presence of damselfish significantly reduced biomass across treatments (Table 3, ANOVA, Herbivory: F_(1,84)_, P < 0.0001), the epilithic algae produced less biomass when grown in the B1 treatment as compared to the A1FI scenario (Table 3, ANOVA, Scenario: F_(2,84)_, P = 0.04). The biomass increase in the absence of herbivory led to almost double the biomass under PD and A1FI conditions, while this effect was less pronounced in the B1 scenario. Whole sample biomass at the end of the experiment (including epi- and endolithic communities, Fig 4) was governed by herbivory and scenario treatment effects and, as with species composition, there was no interaction of herbivory and scenario. When subjected to two future scenarios, the biomass of the whole algal sample was significantly reduced in the B1 treatment compared to the control and A1FI treatments (Table 3, ANOVA, Scenario: F_(2,84)_, P = 0.002), exhibiting a similar trend as the epilithic algal biomass. Also, herbivory led to a significant decrease in whole-sample biomass, irrespective of the scenario treatment (Table 3, ANOVA, Herbivory: F_(1, 84)_, P = 0.0005). The PERMOANOVA analysis performed on all biomass measurements combined confirmed the results of the ANOVAs, where biomass was smaller in samples subjected to herbivory (Herbivory: P(perm) = 0.001) and the biomass was generally reduced under the B1 scenario treatment (Scenario: P(perm) = 0.003).

**Table 3:** Results of the statistical analysis of biomass and productivity data.

Source of variation	Sum of squares	df	Mean square	F	P	Conclusions/*Post hoc* test
turf biomass (g_AFDW_ cm^-2^)						
H	0.136	1	0.136	34.9	<0.0001*	with H < without H
Sc	0.026	2	0.013	3.4	0.039*	B1 < A1FI
H x Sc	0.011	2	0.006	1.4	0.24	
endolithic biomass (g_AFDW_ cm^-2^)						
H	0.002	1	0.002	0.4	0.54	
Sc	0.034	2	0.017	3.9	0.024*	PD > B1 = A1FI
H x Sc	0.004	2	0.002	0.5	0.64	
whole sample biomass (g_AFDW_ cm^-2^)						
H	0.04	1	0.040	13.2	0.0005*	with H < without H
Sc	0.041	2	0.021	6.8	0.0018*	B1 < A1FI = PD
H x Sc	0.006	2	0.003	0.9	0.4	
P_nmax_ (normalized to biomass)						
H	4.882	1	4.882	20.3	<0.0001*	with H < without H
Sc	3.793	2	1.896	7.9	0.0007	B1 > PD = A1FI
H x Sc	0.365	2	0.182	0.8	0.47	
dark respiration (normalized to biomass)						
H	2.918	1	2.918	20.3	<0.0001*	with H < without H
Sc	0.664	2	0.332	2.3	0.11	ns
H x Sc	0.102	2	0.051	0.4	0.7	ns

The data were analyzed using two-way ANOVA for the biomass (turf and endolithic communities as well as the whole sample), and respirometry data (P_nmax_ and dark respiration). ns = not significant,

* = P ≥ 0.05; H = herbivory; Sc = CO_2_ emission scenario; PD = present-day scenario.

### Effects on algal productivity

**Fig 5:**
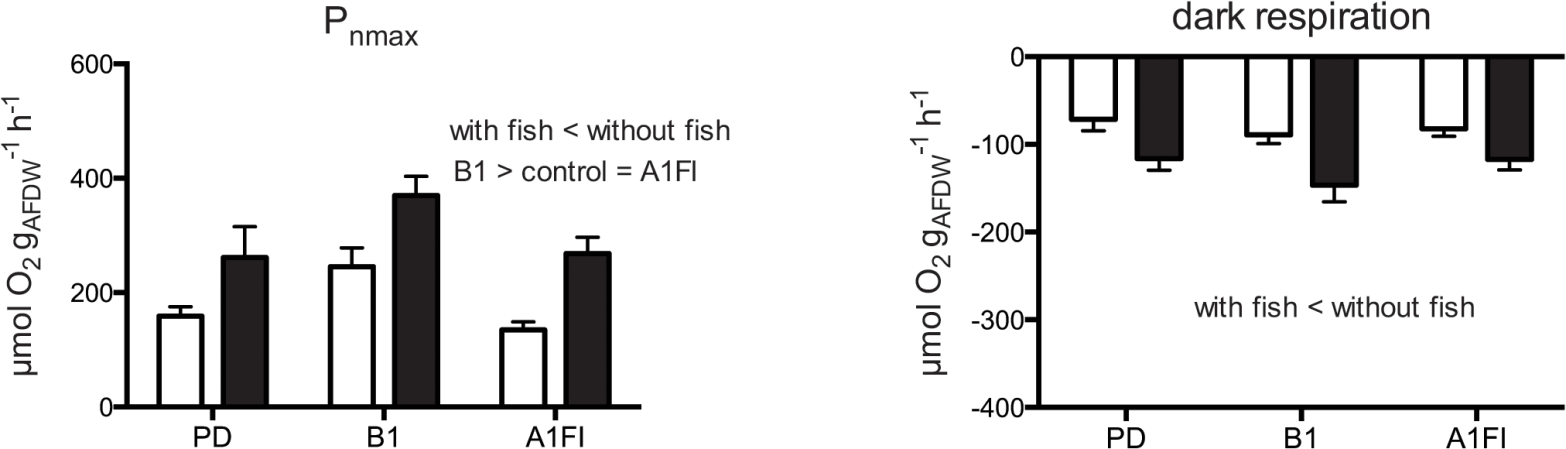
Algal productivity. Respirometry data (P_**nmax**_ = net maximum productivity, μmol O_**2**_ h^-1^ mg^-1^), normalized to whole sample biomass (g_**AFDW**_) under the different CO_**2**_ emission scenarios (B1 = “reduced” and A1FI = “business-as-usual” emission) as well as present-day (PD) conditions. Values are expressed as mean ± SE, white bars indicate presence of farming damselfish, black bars indicate absence of fish, n = 15. The results of the *Post-hoc* analysis are included to highlight significant differences.

The productivity measures of the turf algal communities, measured as O_2_ flux, were affected by farming and scenario treatments, but there was no significant statistical interaction between treatments (Fig 5, Table 3 and confirmed in the PERMANOVA analysis, where Herbivory: P(perm) = 0.0001, and Scenario: P(perm) = 0.0002). Net productivity (normalized to biomass) and dark respiration were reduced when the algae were grazed by damselfish (Table 3, ANOVA, F_(1,84)_, P < 0.0001 for all measures of productivity). Furthermore, P_nmax_ was higher for algae grown in B1 scenarios compared to those in control and A1FI scenarios, irrespective of herbivory (Table 3, ANOVA, Scenario: F_(2,84)_, P = 0.0007 for both productivity measures).

## Discussion

The aim of the present study was to assess how algal turf assemblages farmed by damselfish would be affected in spring by end-of-century SW temperature and acidification scenarios representative of conditions associated with distinct CO_2_ emission through 2050. To this end, algal communities farmed by *P*. *wardi* were subjected to combined elevated SW temperature and increased *p*CO_2_ (B1 and A1FI scenarios) for one month in spring. The results suggest that the presence or absence of the damselfish determines the species composition of algal turfs and that significant impacts of climate change scenario conditions are limited to turf assemblages that are not farmed by fish. However, algae had reduced biomass under the B1 scenario, even in comparison to algae exposed to present-day SW temperature and CO_2_. This observation is counter the expected response as it is generally reported in the literature that climate change will led to a greater abundance of algae on the reef [61]. In the present study, it is demonstrated that efforts to reduce CO_2_ emissions by 2050 (B1) may, through algal biomass reduction, have the positive outcome of limiting phase shifts to algae, an outcome that was not observed under business-as-usual emission (A1FI) scenarios.

The assessment of the algal assemblages farmed by *P*. *wardi* revealed that species composition was dominated by *Polysiphonia* sp. as well as corticated red algae. Similar assemblages have been described in the literature for lower latitudes of the GBR [13,19]. Furthermore, we observed that corticated red algae of the genus *Gelidiella* and non-epiphytic cyanobacteria (mostly Oscillatoriaceae) were more abundant in the presence of *P*. *wardi*. By contrast, the absence of farming was found to promote green and brown algae, specifically *Feldmannia*, *Ulva*, *Cladophora*, *Derbesia* as well as cyanobacteria that were growing epiphytic on eukaryotic turf algae (mostly Rivulariaceae). Some less abundant green and brown algae, namely *Bryobesia johannae* and *Sphacelaria* spp, were also responsible for the differences observed between farmed and non-farmed assemblages as they were excluded by the damselfish. These observed differences were found to be independent of the differences found in community structure at the commencement of the experiment. The fact that assemblages collected from a similar region of the reef flat were significantly different, suggests either that very localized environmental conditions drive these differences, or that the individual fish have different farming practices or both [12,13].

The presence/absence of fish dominated the response of the algal turf community structure. Scenario effects were statistically weak and only observed in the absence of fish. The brown alga *F*. *mitchelliae* and *Ulva* sp. responded negatively, but the green alga *Cladophora* sp. responded positively to B1 and A1FI conditions. *Cladophora* sp. benefitted from the combination of increased temperature and *p*CO_2_, as these may have provided some energy savings in regards to the CCM [37] and might have accelerated its metabolism. *F*. *mitchelliae* and *Ulva* sp. may have been outcompeted and/or been negatively affected by the concurrent increase of temperature and *p*CO_2_. This is not expected considering the positive impacts of elevated CO_2_-only treatments described in the literature [31,32,43–46].

The literature tends to suggest that farming enhances productivity measured as oxygen evolution or biomass [11,14,15]. In the present study, all oxygen flux measurements were negatively influenced by the farming activity of the fish, suggesting that grazing pressure resulted in a loss of tissue and energy for plants. Grazing has been shown to lead to reduced photosynthetic capacity in terrestrial plants [62]. The discrepancy in productivity observed in the present study compared to earlier research may be due to the design of the experiment. In the present experiment, farming reduced net productivity (measured as oxygen flux) in comparison to an un-grazed and un-farmed control. In previous studies that were exclusively field based, the controls were “pavements” of heavily grazed substrate by fish other than damselfish [11,14,15], suggesting that grazing reduces productivity. An effect that is negated by the damselfish’s ability to chase other grazers away and/or by defecation of these territorial damselfish directly over the top of turf farms. Interestingly, ammonium levels were found to be low across tanks in all scenarios and the fish were observed to defecate in the corners of the tank, roughly equidistant from farmed and un-farmed assemblages. These observations suggest that the nutrients were not the root cause for the difference in productivity, although a more definitive experiment is required.

The increase in P_nmax_ relative to biomass under B1 conditions did not carry through to A1FI conditions, where a further temperature and *p*CO_2_ increase returned productivity back to control/present-day conditions. This indicates that the algae were differentially handling further combined increases in SW *p*CO_2_ and temperature. The data suggest that optimal production occurs under the B1 scenario, however, production was measured as O_2_ flux, not carbon fixation *per se*. The distinction between these response variables is clarified by the further observation that under the B1 scenario, biomass (mg_AFDW_ cm^-2^, which is ~80% carbohydrates [63]) is significantly reduced compared to either PD or A1FI scenarios. In order to explain the decreased biomass and increased productivity measured as oxygen flux, the respiration data may be considered. Respiration, however, was constant across all scenarios suggesting that the additional energy under B1 is neither directed to storage, nor growth, but rather is being consumed by additional maintenance costs [64]. The endolithic and the epilithic communities had reduced biomass under B1 scenarios irrespective of farming. Given that there were no significant changes in algal community composition due to scenario treatment in the presence of fish farming, the observed increase in productivity and decrease in biomass in the B1 scenario were most likely directly caused by the combination of elevated temperature and *p*CO_2_ rather than by a shift in algal genera. However, a potential shift in species within genera cannot be ruled out. The reduction in biomass under B1 scenarios indicates that the amount of food available for herbivores may be compromised in the future. If this applies not only to turf algal assemblages but to macroalgal communities in general, potential phase shifts from coral- to algal dominated systems may become less likely under spring B1 scenarios.

The combination of elevated seawater *p*CO_2_ and high temperature under A1FI scenarios led to biomass and productivity levels that were similar to those observed under present-day conditions. Under B1 scenarios, however, biomass was decreased and productivity relative to total biomass increased as a result of warming and acidification. The results suggest that under B1 conditions, either turf algae altered their energy budgets and directed acquired energy away from biomass to other metabolic processes such as maintenance; or greater O_2_ production did not correlate with greater rates of carbon fixation with other mechanics of photochemical quenching making up the difference [64]. Most importantly, the results point to non-linear interactive effects between temperature and acidification on key aspects of turf algal biology [65]. The results emphasize the need to understand the effects of different future scenarios in different seasons to gain an insight into the potential net annual outcome of different future scenarios. The present paper therefore represents a first step in this direction and advocates against using additive linear models to predict future outcomes based on temperature only and acidification only experimental results.

The fish used in this study did not show any visible changes in behavior, nor did they significantly change their selectivity for algae during the course of this experiment. The algal assemblages were dominated by red algae, irrespective of the scenario treatment, and no changes in the relative red algal community composition were observed. It is somewhat surprising that no changes in behavior and selectivity of the fish were observed, as acidification, whilst not modified independently of temperature, reached *p*CO_2_ levels that were comparable to those in studies where fish behavior was observed to be affected (e.g. [47–49]). The differences may be due the fact that (1) temperature modifies the *p*CO_2_ induced response, (2) larvae are more affected than adult fish, (3) the farming behavior is not affected by the physiological and behavioral alterations found in other experiments. However, the findings of the present study could change if the fish had been reared in future conditions. It also cannot be ruled out that changes in olfactory, auditory, or visual function occurred as the fish may have been compensating for a reduction in one sense by upregulating another sense.

In conclusion, this study shows that spring turf algal communities farmed by the damselfish *P*. *wardi* are likely to be stable when subjected to temperature and CO_2_ increases predicted to occur during this century. Farming and grazing by the fish dominated the effects on the composition of these communities and kept it consistent through two different combined elevated temperature and acidity scenarios. However, there was a trend for changes in algal composition with scenario treatment in the absence of the fish, which was species/genus specific. In the B1 scenario increased productivity was paired with decreased biomass irrespective of farming, and it therefore seems like this pairing of temperature and *p*CO_2_ does not promote algal biomass accumulation. This reduction could have implications for herbivorous fish over the long term, including potential reductions in food availability, although system complexities (e.g. due to coral bleaching and other climate change stressors) are such that predictions into the future of algal communities are difficult to make. It is of importance to investigate whether this depression in biomass would carry through and be maintained in summer. In the A1FI scenario, the algae seemed to re-establish productivity and biomass to control levels, yet again the full potential of the A1FI scenario, especially with respect to temperature, would only be reached in a summer experiments. It remains to be tested if the temperature increase would be bearable for the specific algae within fish territories. The results as they stand suggest that a reduced, in contrast to a business-as-usual, CO_2_ emission scenario does not promote algal growth, at least over spring periods. This is an interesting result, especially if it carried through to the majority of coral reef algae, as it may reduce the potential for coral-algal phase shifts in the future.

## Supporting Information

S1 Dataset The experimental raw data. (XLSX)
